# Estimated Incidence of Hospitalisations and Deaths Attributable to Respiratory Syncytial Virus Infections in Adults in Australia Between 2010 and 2019

**DOI:** 10.1111/irv.70092

**Published:** 2025-04-20

**Authors:** Zirke Wiid, Caihua Liang, Robin Bruyndonckx, Lauren Mason, Aleksandra Polkowska‐Kramek, Pimnara Peerawaranun, Mikel Esnaola, Worku Biyadgie Ewnetu, Somsuvro Basu, David Witcombe, Bradford D. Gessner, Elizabeth Begier

**Affiliations:** ^1^ Pfizer Australia Sydney Australia; ^2^ Pfizer Inc. New York City USA; ^3^ P95 Epidemiology & Pharmacovigilance Leuven Belgium; ^4^ Pfizer Inc. Dublin Ireland

**Keywords:** adults, Australia, hospitalisation, mortality, quasi‐Poisson regression, respiratory syncytial virus, time‐series modelling

## Abstract

**Background:**

Respiratory syncytial virus (RSV) morbidity and mortality in adults are often underestimated due to nonspecific symptoms, limited standard‐of‐care testing and lower diagnostic testing sensitivity compared with children. To accurately evaluate the RSV disease burden among adults in Australia, we conducted a model‐based study to estimate RSV‐attributable cardiorespiratory hospitalisation incidence and mortality rate.

**Methods:**

A quasi‐Poisson regression model was used to estimate RSV‐attributable cardiorespiratory, respiratory and cardiovascular events, using weekly hospitalisation and mortality data from 2010 to 2019, accounting for periodic and aperiodic time trends and viral activity and allowing for potential overdispersion. The time‐series model compared the variability in confirmed RSV events alongside variability in all‐cause cardiorespiratory events identified from ICD‐10‐AM codes to estimate the number of RSV‐attributable events, including undiagnosed RSV‐related events.

**Results:**

RSV‐attributable incidence of cardiorespiratory hospitalisations increased with age and was highest among adults ≥ 65 years (329.5–386.6 cases per 100,000 person‐years), nine times higher than in adults 18–64 years. The estimated incidence of RSV‐attributable respiratory hospitalisations in adults ≥65 years (219.7–247.8 cases per 100,000 person‐years) was 35‐fold higher than in adults 18–64 years. RSV‐attributable deaths accounted for 4% to 6% of cardiorespiratory deaths in adults ≥ 65 years, with RSV‐attributable mortality rates ranging from 65.6 to 77.6 deaths per 100,000 person‐years and respiratory mortality rates ranging from 20.3 to 24.0 deaths per 100,000 person‐years, both 70‐fold higher than in adults 18–64 years.

**Conclusions:**

This study identified substantial RSV‐associated morbidity and mortality among Australian adults and is the first study to report RSV‐attributable mortality rates for Australia that account for untested events.

## 1 Introduction

Respiratory syncytial virus (RSV) results in substantial morbidity and mortality burden among adults globally, particularly in older individuals and those with coexisting health conditions [1, 2, 3]. A recent meta‐analysis and modelling study of prospective RSV burden studies estimated that in 2019, RSV caused 787,000 RSV‐related hospitalisations and between 22,000 and 47,000 in‐hospital deaths among older adults in high‐income countries alone [4].

RSV incidence in adults is often underestimated due to overlapping symptoms with other respiratory illnesses, lower viral loads among adults as compared with children, lack of routine testing, exclusion of some RSV cases when using certain illness definitions (e.g. influenza‐like illness or community‐acquired pneumonia), limited diagnostic capabilities and high costs associated with PCR testing [5, 6, 7, 8]. Furthermore, PCR testing using a single respiratory swab has reduced sensitivity in older adults compared with children [8, 9].

A similar challenge of underestimating RSV incidence in adults has been reported in Australia. There is limited national information on RSV incidence and seasonality from prospective studies. RSV testing practices were not uniform before 2021, when RSV became a nationally notifiable disease. A recent study on RSV‐coded hospitalisations revealed substantial differences in RSV testing trends based on patient age. Young children had stable rates of RSV‐attributable hospitalisation between 2006 and 2015, while older adults experienced a 20‐fold increase in RSV‐related hospitalisations during the same period [10], which is likely an artefactual difference due to stable testing in children and increased testing in adults. Another study using multiple regression analysis demonstrated that the highest annual estimates of RSV‐related respiratory hospitalisations (i.e. 464 per 100,000 in children < 5 years and 360 per 100,000 in adults > 75 years) occurred in the extreme age groups. These modelled estimates of RSV hospitalisations were eight times higher than coded hospitalisations in adults ≥ 75 years, indicating the under‐ascertainment/underreporting of adult cases [11], as time‐series modelling can indirectly account for undiagnosed RSV disease events. Alongside these issues in obtaining accurate hospitalisation incidence data, there are limited published data on the true burden of RSV‐associated mortality among older adults in Australia [10, 12].

Because the RSV burden in Australia is substantial, even though not fully weighted, it is vital to prioritise and apply RSV prevention strategies effectively. Two RSV vaccines (Pfizer's Abrysvo [RSVpreF] and GSK's Arexvy [RSVpreF3]) have recently been approved in Australia for adults ≥60 years. Local RSV incidence estimates can enhance understanding of adult disease impact and inform clinical guidelines and reimbursement strategy decisions post‐product registration. In this study, we applied statistical models using data from Australian hospitalisation and mortality databases to estimate the number of RSV‐attributable hospitalisations and deaths from cardiorespiratory diseases among adults from 2010 to 2019.

## 2 Methods

### 2.1 Study Design

This study was an observational retrospective database study to estimate the incidence rates (IRs) of RSV‐attributable cardiorespiratory hospitalisations and mortality among adults in Australia from 2010 to 2019. The estimation was performed using a quasi‐Poisson regression model, adjusting for seasonality and viral activity. The study design was selected following a review of the literature on model‐based estimation of RSV‐attributable IRs using claims or electronic health records databases. The complete generic study protocol that was adapted for this study has been published previously [13].

### 2.2 Study Population and Data Sources

The study population comprised females and males ≥ 18 years residing in Australia. Weekly aggregated hospitalisation data were obtained from the Australian Institute of Health and Welfare (AIHW) National Hospital Morbidity Database. This database, compiled from data supplied by Australian state and territory health authorities, contains anonymised electronic summary records for episodes of care in both public and private Australian hospitals. Weekly aggregated mortality data were obtained from the AIHW National Mortality Database. Data for both hospitalisations and mortality were captured from 2010 to 2019. Individuals were classified into three distinct age groups: 18–64, 65–74 and ≥ 75 years.

All outcomes were identified according to the International Classification of Diseases, tenth revision, Australian modification (ICD‐10‐AM) diagnosis codes. The study focused on three primary disease outcomes: all respiratory diseases (ICD‐10‐AM codes: J00—J99), all cardiovascular diseases (ICD‐10‐AM codes: I00—I99) and all cardiorespiratory diseases (ICD‐10‐AM codes: J00—J99 and I00—I99). Primary and secondary diagnoses were considered for hospitalisation categorisation, while only the underlying cause of death was considered for mortality. A hospitalisation was defined as an overnight stay in the hospital and was characterised by the date of hospital admission.

Influenza and RSV proxies were included in the model as they are the primary time‐varying pathogens responsible for all‐cause respiratory hospitalisation trends. Because RSV testing is more frequent and has higher sensitivity among infants [5, 14, 15, 16], paediatric RSV activity was used as a proxy for RSV, allowing for consistent measurement of RSV activity. Consequently, the RSV proxy was defined as the weekly number of RSV‐related hospitalisations (i.e. with ICD‐10‐AM codes B97.4, J21.0, J12.1, J20.5, J21.9) in children < 2 years. Following a similar rationale, because influenza testing is most consistently conducted among older adults [17] and the burden is highest among older adults, the influenza proxy was defined as the weekly number of influenza‐specific hospitalisations (i.e. with ICD‐10‐AM codes J09, J10, J11) in adults ≥ 65 years.

To assess the underestimation in recorded (reported based on standard‐of‐care testing) RSV events when compared with attributable (model‐based) RSV events, we also obtained the annual number of RSV‐specific hospitalisations (ICD‐10‐AM codes B97.4, J21.0, J21.1, J20.5).

### 2.3 Statistical Model

Outcomes (i.e. weekly hospitalisations or deaths) displaying a seasonal pattern were modelled with a quasi‐Poisson regression model stratified by age group. All models were fitted as described in the published time‐series model‐based analysis protocol [13].

In brief, the weekly number of events was modelled, accounting for periodic and aperiodic time trends and viral activity and allowing for potential overdispersion. A polynomial up to the fourth order was used to capture the aperiodic time trend in the weekly number of events. Yearly and half‐yearly harmonic terms with weekly periodicity (period = 52.143) were used to reflect the seasonal variation in the weekly number of events. To reduce flexibility and avoid overfitting for the mortality data, which show substantially less variability than the hospitalisation data, the model for mortality included only the yearly harmonic terms.

RSV and influenza proxies lagged by 0–4 weeks were considered as community viral activity proxies. Other potentially relevant pathogens contributing to cardiorespiratory, respiratory and cardiovascular hospitalizations and deaths are indirectly accounted for through the periodic component and overdispersion parameter. The identity link function was used to reflect the most biologically plausible link between viral circulation and the occurrence of events.

The resulting fitted models were used to obtain the annual number of RSV‐attributable hospitalisations and deaths for each considered outcome and age group. We obtained yearly age‐specific IRs by dividing the annual number of RSV‐attributable events by the corresponding number of at‐risk individuals. The number of at‐risk individuals was derived from Australian census data from the Australian Bureau of Statistics. To reflect the changes in the population as accurately as possible, the number of at‐risk individuals was composed by combining the preceding and subsequent censuses. For the 2010–2011 period, the 2011 census was used, while for 2012–2015, the average between the 2011 and 2016 censuses was used. For 2016, the 2016 census was used, and for 2017–2019, the average between the 2016 and 2019 censuses was used. The proportions of RSV‐attributable events were calculated by dividing the annual number of RSV‐attributable events by the annual number of observed events.

The annual IRs of RSV‐specific hospitalisations per age group were calculated using RSV‐specific hospitalisations data and were compared with the model‐based IRs of RSV‐attributable events.

All statistical analyses were performed using R version 4.2.2.

### 2.4 Ethics

All data used in the execution of the study were aggregated and anonymised secondary data and obtained with the approval of the data owner. Consequently, no Institutional Review Boards/independent ethics committee approval was required. Informed consent from patients was not needed due to using anonymised structured data with no patient personal information. The study was conducted per legal and regulatory requirements and adhered to scientific value and rigour based on the International Epidemiological Association's Good Epidemiological Practice guidelines.

## 3 Results

### 3.1 Study Population

During the study period of 2010–2019, there were a total of 11.7 million cardiorespiratory hospitalisations in adults ≥ 18 years in Australia, of which 8.8 million were coded with a cardiovascular disease and 4.7 million were coded with a respiratory disease (note that a patient may be hospitalised for both a cardiovascular and a respiratory disease simultaneously). In the same period, 581,169 cardiorespiratory deaths occurred, of which approximately 75% were identified as cardiovascular and 25% were identified as respiratory deaths. The majority of hospitalisations and deaths occurred in those ≥ 75 years.

### 3.2 Estimated RSV‐Attributable Hospitalisations

The estimated annual IRs of RSV‐attributable cardiorespiratory, respiratory and cardiovascular hospitalisations are presented in Table 1. RSV‐attributable hospitalisations represented between 1.3% and 2.4% of total cardiorespiratory hospitalisations in each year and age group. The IRs varied slightly over the study period, with the highest rates observed in 2015 among age groups ≥ 65 years. IRs increased with age, with estimated RSV‐attributable cardiorespiratory hospitalisations ranging from 37.8 to 44.7 cases per 100,000 person‐years in adults 18–64 years and increasing considerably in adults ≥ 65 years (329.5–386.6 cases per 100,000 person‐years), with the highest rates observed in those ≥ 75 years (418.0–480.1 cases per 100,000 person‐years). Estimated cardiorespiratory hospitalisations in adults ≥ 65 years were approximately nine‐fold higher compared with adults 18–64 years.

**TABLE 1:** Estimated incidence rate (IR) per 100,000 person‐years of RSV‐attributable hospitalisations and percentage (%) of hospitalisations attributable to RSV, stratified by age group.

Year	18–64 years		65–74 years		≥ 75 years		≥ 65^†^ years	
	%	IR	%	IR	%	IR	%	IR
		[95% CI]		[95% CI]		[95% CI]		
RSV‐attributable cardiorespiratory hospitalisations								
2010	1.4	38.1	2.4	305.4	1.4	446.0	1.7	370.0
		[0–153.8]		[0–844.4]		[0–1344.9]		
2011	1.4	38.2	2.3	306.2	1.4	447.2	1.7	371.0
		[0–154.2]		[0–846.6]		[0–1348.4]		
2012	1.4	37.8	2.2	276.0	1.3	425.7	1.6	342.8
		[0–152.4]		[0–763.3]		[0–1283.7]		
2013	1.4	37.8	2.1	276.1	1.3	425.8	1.6	342.9
		[0–152.5]		[0–763.5]		[0–1284.0]		
2014	1.4	38.9	2.1	284.2	1.3	438.3	1.5	352.9
		[0–156.9]		[0–785.8]		[0–1321.6]		
2015	1.5	42.6	2.2	311.3	1.3	480.1	1.6	386.6
		[0–171.9]		[0–860.8]		[0–1447.7]		
2016	1.4	40.6	2.1	274.0	1.3	442.0	1.6	347.1
		[0–163.8]		[0–757.6]		[0–1332.7]		
2017	1.4	40.8	2.1	260.9	1.3	418.0	1.6	329.5
		[0–164.6]		[0–721.4]		[0–1260.4]		
2018	1.4	41.5	2.1	265.8	1.3	425.8	1.6	335.7
		[0–167.7]		[0–735.0]		[0–1284.0]		
2019	1.6	44.7	2.3	286.1	1.4	458.3	1.7	361.3
		[0–180.5]		[0–791.1]		[0–1382.1]		
RSV‐attributable respiratory hospitalisations								
2010	0.6	6.5	2.6	114.8	3.5	402.0	3.2	246.8
		[0–56.6]		[0–265.8]		[50.9–783.3]		
2011	0.5	6.5	2.4	115.1	3.3	403.9	3.0	247.8
		0–56.7]		[0–266.5]		[51.1–786.9]		
2012	0.5	6.5	2.3	103.7	3.1	384.6	2.8	229.1
		[0–56.1]		[0–240.3]		[48.7–749.5]		
2013	0.5	6.5	2.3	103.8	3.2	385.1	2.9	229.3
		0–56.1]		[0–240.4]		[48.7–750.5]		
2014	0.5	6.6	2.1	106.8	3.1	394.9	2.8	235.3
		[0–57.7]		[0–247.4]		[50.0–769.4]		
2015	0.6	7.3	2.2	117.0	3.1	435.3	2.8	259.0
		[0–63.2]		[0–271.0]		[55.1–848.2]		
2016	0.5	6.9	2.0	103.0	3.0	398.1	2.7	231.4
		[0–60.2]		[0–238.5]		[50.4–775.7]		
2017	0.5	7.0	2.0	98.1	2.8	376.4	2.5	219.7
		[0–60.6]		[0–227.1]		[47.6–733.5]		
2018	0.6	7.1	2.1	99.9	3.1	385.2	2.8	224.5
		[0–61.7]		[0–231.4]		[48.7–750.6]		
2019	0.6	7.6	2.2	107.5	3.2	414.1	2.8	241.5
		[05–66.4]		[0–249.1]		[52.4–807.0]		
RSV‐attributable cardiovascular hospitalisations								
2010	—	—	—	—	1.0	276.5	—	—
						[0–1076.6]		
2011	—	—	—	—	1.0	277.2	—	—
						[0–1079.4]		
2012	—	—	—	—	1.0	263.9	—	—
						[0–1027.6]		
2013	—	—	—	—	1.0	263.9	—	—
						[0–1027.9]		
2014	—	—	—	—	1.0	271.7	—	—
						[0–1058.0]		
2015	—	—	—	—	1.0	297.6	—	—
						[07–1158.9]		
2016	—	—	—	—	1.0	274.0	—	—
						[0–1066.8]		
2017	—	—	—	—	1.0	259.1	—	—
						[01–1008.9]		
2018	—	—	—	—	1.0	263.9	—	—
						[0–1027.9]		
2019	—	—	—	—	1.1	284.1	—	—
						[0–1106.3]		

*Note:* Negative estimates were suppressed to zero because of biological implausibility.

† Data for the ≥65 years age group were not modelled but based on pooling across age groups. Therefore, confidence intervals are not presented for this age group.

Similarly, estimated RSV‐attributable respiratory hospitalisations were also highest among older adults, with rates in the ≥ 65 years age group (219.7–259.0 cases per 100,000 person‐years) 35‐fold higher than those in adults 18–64 years (6.5–7.6 cases per 100,000 person‐years). RSV‐attributable cardiovascular hospitalisations were only modelled for the oldest age group, as data were randomly distributed without a clear seasonal pattern in the younger age groups. IRs for estimated RSV‐attributable cardiovascular hospitalisations were lower than that of the estimated RSV‐attributable cardiorespiratory and respiratory hospitalisations in adults ≥ 75 years, ranging from 259.1 to 297.6 cases per 100,000 person‐years.

### 3.3 Estimated RSV‐Attributable Deaths

The estimated RSV‐attributable mortality rates for cardiorespiratory, respiratory and cardiovascular deaths are presented in Table 2. RSV‐attributable deaths represented between 2.1% and 2.7% of total cardiorespiratory deaths in adults 18–64 years and between 4.3% and 5.6% of cardiorespiratory deaths in adults ≥ 65 years. Mortality rates increased with age, with estimated cardiorespiratory deaths ranging from 0.9 to 1.1 cases per 100,000 person‐years in adults 18–64 years and increasing sharply in adults ≥ 65 years, ranging from 65.6 to 77.6 cases per 100,000 person‐years. The highest rates were observed in those ≥ 75 years, ranging from 121.2 to 140.4 cases per 100,000 person‐years. Estimated cardiorespiratory deaths in adults ≥ 65 years were approximately 70‐fold higher than cardiorespiratory deaths in adults < 65 years. Likewise, RSV‐attributable respiratory deaths were 70‐fold greater in adults ≥ 65 years (20.3–24.0 cases per 100,000 person‐years) compared with adults < 65 years (0.3 cases per 100,000 person‐years).

**TABLE 2:** Estimated mortality rate (MR) per 100,000 person‐years of RSV‐attributable deaths and percentage (%) of deaths attributable to RSV, stratified by age group.

Year	18–64 years		65–74 years		≥75 years		≥65 years^†^	
	%	MR	%	MR	%	MR	%	MR
		[95% CI]		[95% CI]		[95% CI]		
RSV‐attributable cardiorespiratory deaths								
2010	2.1	0.9	6.2	26.4	4.1	130.1	4.4	74.1
		[0–2.7]		[6.9–43.6]		[60.9–202.8]		
2011	2.1	0.9	6.3	26.3	4.0	129.7	4.3	73.9
		[0–2.7]		[6.9–43.5]		[60.7–202.1]		
2012	2.3	0.9	6.5	24.0	4.1	125.0	4.4	69.1
		[0–2.7]		[6.3–39.7]		[58.5–194.9]		
2013	2.2	0.9	6.4	24.0	4.3	124.6	4.6	68.9
		[0–2.7]		[6.3–39.6]		[58.3–194.3]		
2014	2.2	0.9	6.2	24.5	4.2	127.5	4.5	70.5
		[0–2.8]		[6.4–40.5]		[59.7–198.8]		
2015	2.4	1.0	6.7	27.0	4.5	140.4	4.8	77.6
		[0–3.0]		[7.0–44.6]		[65.7–218.8]		
2016	2.4	1.0	6.6	23.7	4.6	128.8	4.8	69.4
		[0–2.9]		[6.2–39.1]		[60.3–200.7]		
2017	2.4	1.0	6.4	22.4	4.5	121.2	4.8	65.6
		[01–2.9]		[5.9–37.0]		[56.7–188.9]		
2018	2.5	1.0	6.9	23.1	5.1	124.8	5.4	67.5
		[0–3.0]		[6.0–38.1]		[58.4–194.4]		
2019	2.7	1.1	7.4	24.7	5.3	133.5	5.6	72.3
		[0–3.2]		[6.5–40.8]		[62.5–208.1]		
RSV‐attributable respiratory deaths								
2010	3.7	0.3	4.7	5.2	6.7	43.8	6.4	22.9
		[0–1.1]		[0–15.6]		[12.2–74.8]		
2011	3.6	0.3	4.5	5.2	6.4	43.6	6.1	22.9
		[0–1.1]		[0–15.7]		[12.1–74.5]		
2012	3.7	0.3	4.4	4.7	6.1	42.1	5.8	21.4
		[0–1.1]		[0–14.1]		[11.7–71.9]		
2013	3.8	0.3	4.3	4.7	6.7	41.9	6.3	21.3
		[0–1.1]		[0–14.2]		[11.7–71.6]		
2014	3.4	0.3	3.9	4.8	6.2	42.9	5.8	21.8
		[0–1.1]		[0–14.5]		[11.9–73.3]		
2015	3.7	0.3	4.3	5.3	6.5	47.2	6.1	24.0
		[0–1.3]		[0–16.0]		[13.1–80.7]		
2016	3.7	0.3	4.2	4.7	6.4	43.3	6.0	21.5
		[0–1.2]		[0–14.0]		[12.1–74.0]		
2017	3.4	0.3	3.9	4.4	5.9	40.8	5.5	20.3
		[0–1.2]		[0–13.3]		[11.3–69.6]		
2018	3.8	0.3	4.4	4.5	6.9	42.0	6.5	20.9
		[0–1.2]		[0–13.6]		[11.7–71.7]		
2019	3.7	0.3	4.5	4.9	6.7	44.9	6.3	22.4
		[−0.7–1.3]		[0–14.7]		[12.5–76.7]		
RSV‐attributable cardiovascular deaths								
2010	—	—	7.1	22.3	4.4	113.2	4.8	64.0
				[7.9–36.1]		[59.5–170.3]		
2011	—	—	7.3	22.2	4.4	113.3	4.8	64.1
				[7.9–36]		[59.6–170.5]		
2012	—	—	7.7	20.2	4.6	108.6	5.0	59.7
				[7.2–32.9]		[57.1–163.4]		
2013	—	—	7.6	20.2	4.7	108.5	5.1	59.6
				[7.2–32.8]		[57.0–163.3]		
2014	—	—	7.6	20.7	4.7	111.2	5.1	61.1
				[7.4–33.5]		[58.5–167.4]		
2015	—	—	8.0	22.7	5.1	122.4	5.5	67.2
				[8.1–36.9]		[64.3–184.2]		
2016	—	—	8.0	19.9	5.2	111.9	5.6	60.0
				[7.1–32.3]		[58.8–168.5]		
2017	—	—	8.0	18.9	5.4	105.8	5.7	56.8
				[6.7–30.7]		[55.6–159.2]		
2018	—	—	8.4	19.5	5.9	108.6	6.2	58.4
				[6.9–31.6]		[57.1–163.5]		
2019	—	—	9.1	20.8	6.4	116.4	6.8	62.6
				[7.4–33.8]		[61.2–175.1]		

*Note:* Negative estimates were suppressed to zero because of biological implausibility.

† Data for the ≥ 65 years age group were not modelled but based on pooling across age groups. Therefore, confidence intervals are not presented for this age group.

RSV‐attributable mortality rates for cardiovascular causes were similarly highest among the eldest patients, although they were not modelled for the youngest age group due to a lack of a clear seasonal distribution. The mortality rates for cardiovascular diseases were more than double that of the rates for respiratory diseases in the ≥ 75 years age group and approximately five times higher for the 65–74‐year age group.

### 3.4 Recorded RSV‐Specific Hospitalisations

**FIGURE 1:**
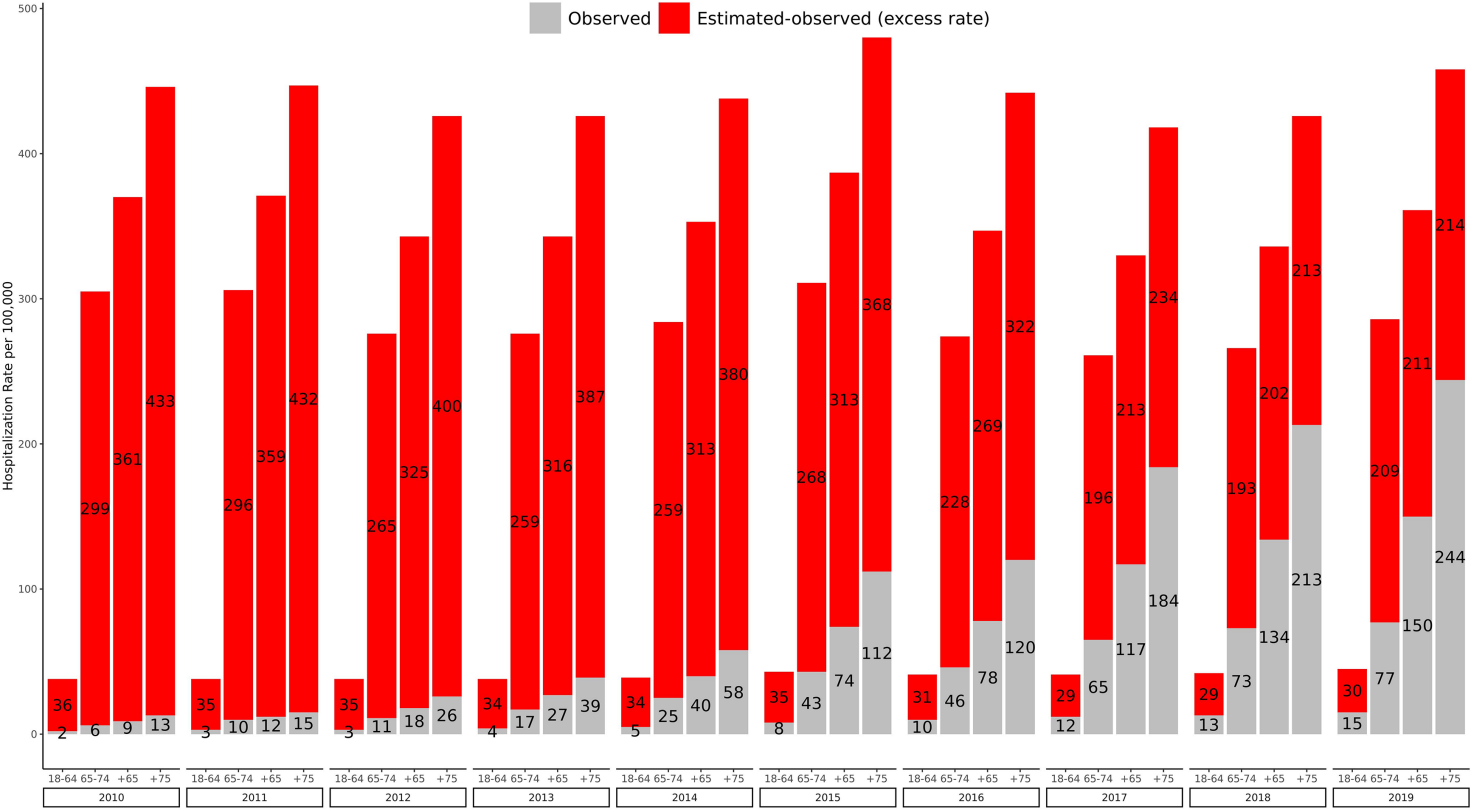
Annual differences between observed diagnosed RSV‐specific hospitalisation rates and estimated RSV‐attributable cardiorespiratory hospitalisation rates, stratified by age group.

The differences between the annual recorded IRs of hospitalisations that were coded as RSV‐specific (i.e. standard‐of‐care diagnoses) and the estimated RSV‐attributable IRs of cardiorespiratory hospitalisations per age group are presented in Figure 1. The recorded RSV‐specific IRs increased over time in all age groups, with the lowest rates reported in 2010 (1.9 and 9.2 cases per 100,000 person‐years in adults 18–64 years and ≥ 65 years, respectively) and the highest rates in 2019 (14.6 and 149.6 cases per 100,000 person‐years in adults 18–64 years and ≥ 65 years, respectively). The magnitude of this increase in IR during the study period was greater with increasing age, with observed rates rising 7.5 times in the 18–64‐years group, 12.8 times in the 65–74‐years group and 18.8 times in the ≥75‐years group, suggesting standard‐of‐care RSV testing and diagnosis increased more among the oldest age groups.

Estimated RSV‐attributable cardiorespiratory hospitalisation rates were substantially higher than recorded RSV‐specific hospitalisation rates. The ratios between the estimated RSV‐attributable IRs and recorded RSV‐specific IRs are presented in Table 3. On average, the estimated RSV‐attributable cardiorespiratory hospitalisation rates in adults 18–64 years and ≥ 65 years were eight and 13 times greater than recorded RSV‐specific hospitalisation rates. Estimated RSV‐attributable respiratory hospitalisation rates were, on average, 1.4 times and nine times greater than recorded RSV‐specific rates in adults 18–64 years and ≥ 65 years, respectively. The differences decreased over the years as recorded RSV‐specific hospitalisations increased. However, in 2019, estimated RSV‐attributable cardiorespiratory hospitalisation rates in adults 18–64 years and ≥ 65 years were still approximately three‐ and two‐fold higher than reported RSV‐specific rates. From 2015 onwards, reported RSV‐specific hospitalisation rates surpassed estimated RSV‐attributable respiratory hospitalisation rates in adults aged 18–64 years. However, in adults ≥ 65 years, estimated RSV‐attributable respiratory hospitalisation rates were still 1.6 times higher than the observed diagnosed rates in 2019.

**TABLE 3:** Annual ratio between model‐based RSV‐attributable IR and observed diagnosed RSV‐specific IR, stratified by age group (years).

Year	Cardiorespiratory hospitalisations				Respiratory hospitalisations			
	18–64	65–75	> = 75	> = 65	18–64	65–75	≥ 75	≥ 65
2010	19.0	50.8	34.3	41.1	3.5	19.2	30.9	27.4
2011	12.7	30.6	29.8	30.9	2.3	11.5	26.9	20.7
2012	12.7	25.1	16.4	19.1	2.0	9.5	14.8	12.7
2013	9.5	16.2	10.9	12.7	1.5	6.1	9.9	8.5
2014	7.8	11.4	7.6	8.8	1.4	4.3	6.8	5.9
2015	5.4	7.2	4.3	5.2	0.9	2.7	3.9	3.5
2016	4.1	6.0	3.7	4.4	0.7	2.2	3.3	3.0
2017	3.4	4.0	2.3	2.8	0.6	1.5	2.0	1.9
2018	3.2	3.6	2.0	2.5	0.5	1.4	1.8	1.7
2019	3.0	3.7	1.9	2.4	0.5	1.4	1.7	1.6

## 4 Discussion

We found a high burden of RSV‐attributable respiratory and cardiorespiratory hospitalisations and mortality among adults in Australia. The incidence and mortality rates increased with age and were highest among adults ≥ 75 years. Consistent with other studies, we observed substantial under‐ascertainment/reporting of RSV cases in all age groups [11, 18]. It is the first study to report RSV‐attributable mortality rates for Australia that account for untested events.

RSV is a common cause of viral acute lower respiratory infections and a common cause of exacerbation of chronic respiratory conditions [2, 19]. The RSV‐attributable respiratory hospitalisation rates in people ≥ 65 years obtained in our study were comparable to those reported in Spain (257–280 hospitalisations per 100,000 person‐years) [18] and Germany (236–363 hospitalisations per 100,000 person‐years) [20]. They are also consistent with the pooled estimates of hospitalisation incidence from prospective studies in the US (282 per 100,000) and globally (347 per 100,000) when adjusted for diagnostic testing based on under‐ascertainment [4, 21]. Our estimate for RSV‐attributable respiratory hospitalisations in adults ≥ 75 years (376–435 hospitalisations per 100,000 person‐years) was slightly higher than the point estimate obtained by the previous model‐based study in Australia (360 cases per 100,000 persons) [11]. A potential reason is that we used primary and secondary diagnosis codes, compared with only primary diagnosis codes in the previous study, to increase the sensitivity of our outcome definitions [22]. The previous study [11] used RSV and influenza hospitalisations in children < 5 years, whereas we used RSV‐related hospitalisations in children < 2 years and influenza‐specific hospitalisations in older adults for proxy measures of activity of each virus, respectively. Also, we used a slightly different definition of RSV proxy by including bronchiolitis unspecified code (J21.9) in children < 2 years. Most bronchiolitis is caused by RSV [23, 24, 25] in this age group and may not be specifically coded as RSV. The close correspondence of the seasonality of unspecified bronchiolitis with RSV bronchiolitis hospitalisations supports this association [10].

Our findings contribute to a growing body of evidence supporting the contribution of RSV to cardiorespiratory hospitalisations [26]. We estimated that approximately 1%–2% of cardiorespiratory hospitalisations in adults 65–74 and ≥ 75 years could be attributed to RSV. Comparable attributable proportions were observed in model‐based studies in the UK and Germany [5, 20]. However, the IRs were higher in our study than in the study from the UK and lower than in the German study. This difference could be related to differences in coding practices, the prevalence of chronic cardiorespiratory conditions between the countries, or model inputs.

Until 2021, RSV was not a notifiable disease in Australia. On average, the RSV‐coded hospitalisation rates in people 18–65 years and ≥65 years were eight and 13 times lower than the IR of RSV‐attributable hospitalisations obtained from our model. However, during the study period, we observed a substantial increase in standard‐of‐care RSV diagnosis rates from 2 to 15 and 9 to 150 cases per 100,000 person‐years in people 18–64 and ≥ 65 years, respectively. This resulted in decreasing ratios between modelled RSV‐attributable and observed RSV diagnosis rates over the years because more RSV‐related events were diagnosed due to increased testing. This observation has been reported previously [10] and might be related to increased awareness and testing for RSV in adults, particularly in older adults [27], as well as increased use of multiplex PCR testing panels instead of single PCR tests for respiratory viruses. The modelled RSV‐attributable rates remain relatively stable over time and align more closely with the observed RSV incidence in the last study years as testing increases, supporting the robustness of our estimates.

We found the highest mortality rates among people ≥ 75 years. We estimated that approximately 6% and 5% of all respiratory and cardiorespiratory deaths in people ≥ 65 years can be attributed to RSV. Our RSV‐attributable respiratory mortality rates in this age group (21–24 deaths per 100,000) were slightly higher than those reported in Spain (14.6–17.1 deaths per 100,000) [18], Italy (12–18 deaths per 100,000) [28], Norway (11–19 deaths per 100,000) [29] and the USA (14.7 deaths per 100,000) [30]. This could potentially be related to differences in coding practices on death certificates. Relatively high and stable mortality rates observed throughout the study period, especially in older adults, highlight the need for prevention strategies, such as vaccination, to reduce excess deaths.

Our study was characterised by several strengths. First, we included national databases. Second, we performed the time series modelling study following a standardised generic protocol based on best practices from past studies [13], which permits robust comparison of results between countries. Furthermore, we included three broad categories of outcomes to better reflect the potential impact of RSV on the hospitalisation and mortality burden.

Several limitations should be taken into account. In our study, we analysed only hospitalisations, which likely underestimated the total burden of RSV as most patients are treated in outpatient settings [31, 32]. Due to data limitations, we could not stratify data by the presence of comorbidities or Indigenous status, which are known factors for a higher risk of severe outcomes [3, 10]. We could also not stratify data by region and RSV seasonality [33, 34]. The model limitations are described in depth in the generic protocol [13].

In conclusion, our study indicates high morbidity and mortality related to RSV in adults in Australia, especially in those ≥ 65 years. Although testing and recognition of RSV have improved over the years, the true burden of RSV in older adults is still underestimated, especially when considering its impact on cardiorespiratory diseases.

## Author Contributions

Bradford D. Gessner and Elizabeth Begier conceived the study. Zirke Wiid, Caihua Liang, Robin Bruyndonckx, Aleksandra Polkowska‐Kramek and Elizabeth Begier designed the study and its statistical analysis. Pimnara Peerawaranun, Mikel Esnaola, Worku Biyadgie Ewnetu, Robin Bruyndonckx, Lauren Mason and Aleksandra Polkowska‐Kramek conducted the analysis and investigation. Lauren Mason, Robin Bruyndonckx, Aleksandra Polkowska‐Kramek, Somsuvro Basu and Mikel Esnaola wrote the manuscript, with Zirke Wiid, Caihua Liang, David Witcombe, Bradford D. Gessner and Elizabeth Begier providing critical feedback and revisions. All authors reviewed and approved the final version of the manuscript.

## Conflicts of Interest

Robin Bruyndonckx, Lauren Mason, Aleksandra Polkowska‐Kramek, Pimnara Peerawaranun, Mikel Esnaola, Worku Biyadgie Ewnetu and Somsuvro Basu are employees of P95 Epidemiology & Pharmacovigilance, which received funding from Pfizer in connection with the development of this manuscript and the research described in this manuscript. Zirke Wiid, Caihua Liang, David Witcombe, Bradford D. Gessner and Elizabeth Begier are Pfizer employees and may own Pfizer stock.

## Data Availability

The data used in this study were provided by the AIHW and are not publicly available. The datasets cannot be provided by the authors upon request due to restrictions from the data provider. However, the datasets can be requested from AIHW using their data request process.
